# Supplementary material for: Design of a Gene Panel to Expose the Versatile Role of Hepatic Stellate Cells in Human Liver Fibrosis
Source: Pharmaceutics. 2020 Mar 20;12(3):278. doi: 10.3390/pharmaceutics12030278 (PMC7151042; doi:10.3390/pharmaceutics12030278)
Supplement: Supplementary file 1 [file pharmaceutics-12-00278-s001.pdf]

pharmaceutics

MDPI

Supplementary Materials: Design of a Gene Panel to Expose the Versatile Role of Hepatic Stellate Cells in Human Liver Fibrosis

Fransien van Dijk, Christa M. Hazelhoff, Eduard Post, Gerian G.H. Prins, Krista Rombouts, Klaas Poelstra, Peter Olinga and Leonie Beljaars

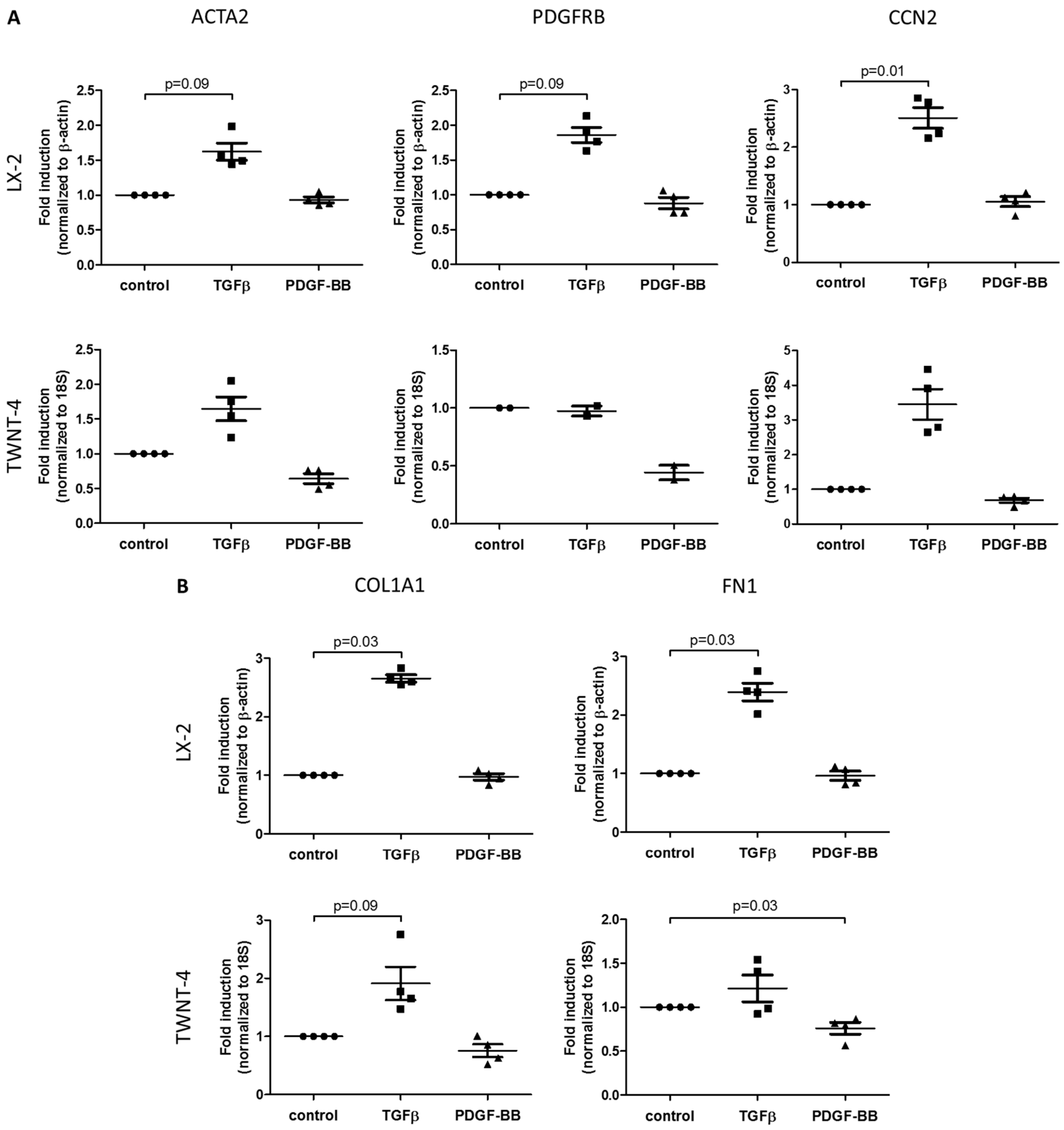

C

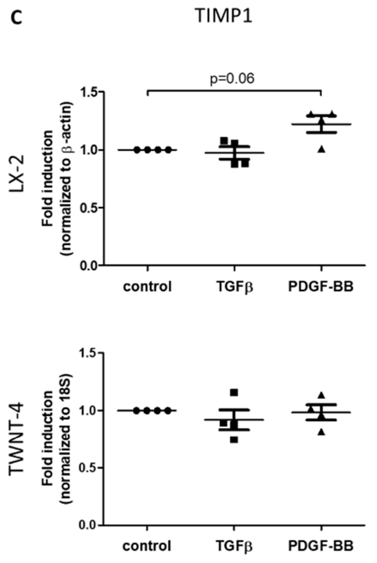
TIMP1
LX-2
Fold induction (normalized to β-actin)
p=0.06
TWNT-4
Fold induction (normalized to 18S)
0.0
0.5
1.0
1.5
control
TGFβ
PDGF-BB

D

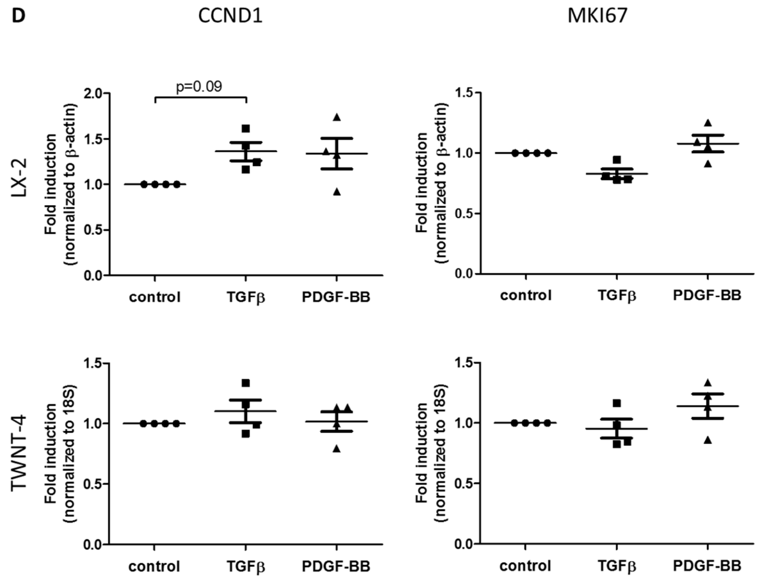
CCND1
MKI67
LX-2
Fold induction (normalized to β-actin)
p=0.09
TWNT-4
Fold induction (normalized to 18S)
0.0
0.5
1.0
1.5
2.0
control
TGFβ
PDGF-BB

E

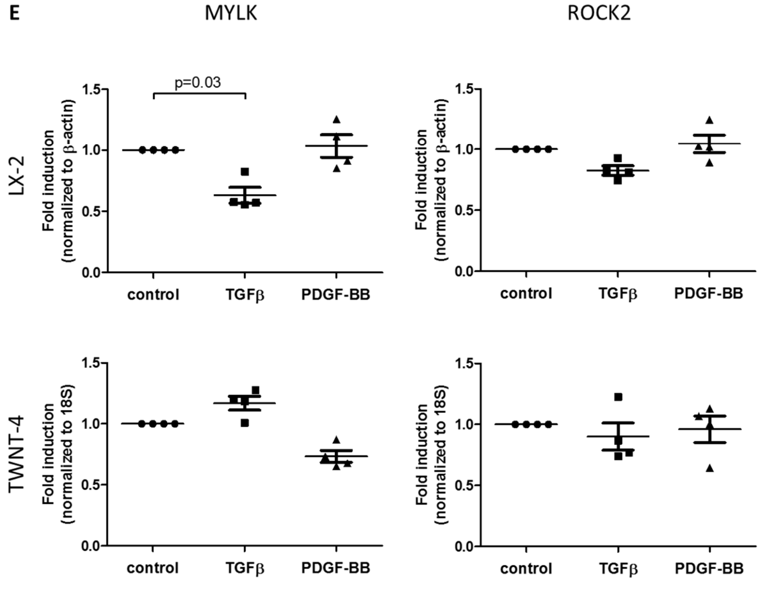
MYLK
ROCK2
LX-2
Fold induction (normalized to β-actin)
p=0.03
TWNT-4
Fold induction (normalized to 18S)
0.0
0.5
1.0
1.5
control
TGFβ
PDGF-BB

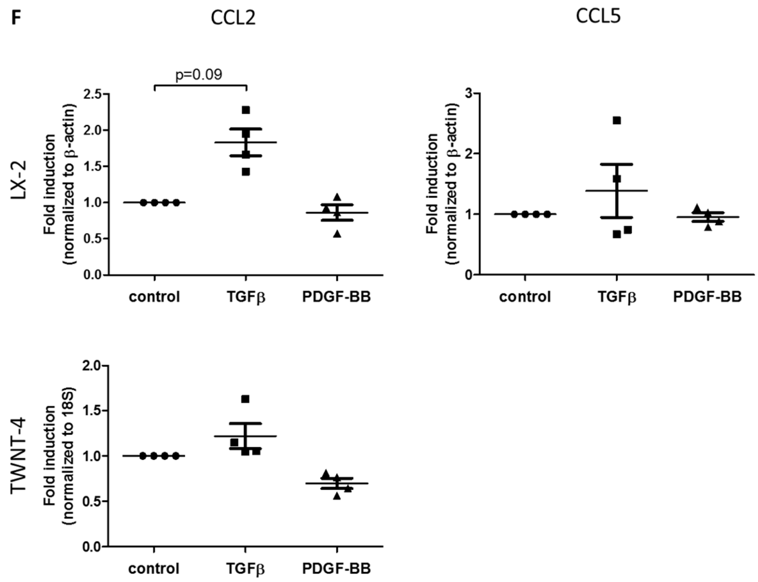

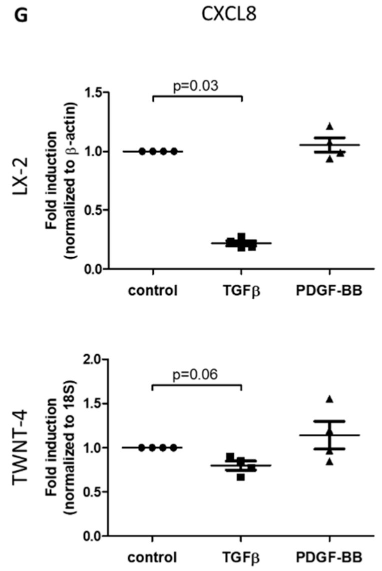

Figure S1. In vitro effects of TGFβ or PDGF-BB on the gene expression of markers for HSC-activities during fibrosis in LX-2 and TWNT-4 cells (both $n = 4$). HSC-activities and their associated genes include (**A**) activation (*ACTA2, PDGFRB, CCN2*), (**B**) fibrogenesis (*COL1A1, FN1*), (**C**) altered matrix degradation (*TIMP1*), (**D**) proliferation (*CCND1, MKI67*), (**E**) contractility (*MYLK, ROCK2*), (**F**) chemotaxis (*CCL2, CCL5*) and (**G**) inflammatory signaling (*CXCL8*). The Ct values of *CCL5* in TWNT-4 cells and *TNF, PPARG* and *CYGB* in both cell types were not detectable. Fold inductions are relative to untreated controls.

Table S1. Ct values ± SEM per gene of the untreated controls in all used in vitro models as determined with quantitative real-time PCR. ND indicates not detectable values.

Gene	Primary HSC	HHSteC	LX-2	TWNT-4	PCLS
ACTA2	19.2 ± 0.1	22.3 ± 0.7	22.0 ± 0.7	25.3 ± 0.3	26.8 ± 0.6
PDGFRB	26.9 ± 0.2	26.8 ± 0.2	29.8 ± 0.6	ND	28.6 ± 0.4
CCN2	18.7 ± 0.1	20.7 ± 0.7	21.7 ± 0.3	22.9 ± 0.2	26.8 ± 0.6
COL1A1	17.2 ± 0.3	18.0 ± 0.1	19.1 ± 0.3	20.3 ± 0.2	22.8 ± 0.4
FN1	17.8 ± 0.2	18.0 ± 0.6	20.7 ± 0.3	22.6 ± 0.3	20.6 ± 0.6
TIMP1	17.6 ± 0.1	19.0 ± 0.2	22.4 ± 0.3	23.2 ± 0.1	20.7 ± 0.2
CCND1	18.7 ± 0.1	22.2 ± 0.1	23.1 ± 0.3	22.5 ± 0.3	24.1 ± 0.5

MKI67	24.2 ± 0.1	24.2 ± 0.7	22.5 ± 0.4	24.5 ± 0.3	28.8 ± 1.1
MYLK	24.2 ± 0.1	25.4 ± 0.2	24.4 ± 0.3	28.4 ± 0.4	27.2 ± 1.2
ROCK2	23.7 ± 0.1	24.9 ± 0.2	24.2 ± 0.4	26.5 ± 0.3	25.5 ± 0.5
CCL2	23.1 ± 0.1	23.2 ± 0.4	30.1 ± 0.3	26.3 ± 0.4	23.7 ± 0.3
CCL5	ND	31.1 ± 1.1	30.6 ± 0.4	ND	27.3 ± 0.4
TNF	ND	ND	ND	ND	29.7 ± 0.7
CXCL8	28.4 ± 0.2	26.4 ± 1.0	29.1 ± 0.6	26.3 ± 0.4	24.9 ± 0.7
PPARG	ND	31.1 ± 0.8	ND	ND	28.9 ± 0.3
CYGB	27.3 ± 0.2	24.7 ± 0.7	ND	ND	29.4 ± 1.1
ACTB	17.1 ± 0.1	16.9 ± 0.1	16.8 ± 0.6	20.5 ± 0.5	19.4 ± 0.2
RNA18S5	10.0 ± 0.2	7.8 ± 0.2	9.4 ± 0.6	13.7 ± 0.6	8.5 ± 0.7
